# Allyl-Isothiocyanate and Microcystin-LR Reveal the Protein Phosphatase Mediated Regulation of Metaphase-Anaphase Transition in *Vicia faba*

**DOI:** 10.3389/fpls.2018.01823

**Published:** 2018-12-13

**Authors:** Tamás Garda, Zoltán Kónya, Csongor Freytag, Ferenc Erdődi, Sándor Gonda, Gábor Vasas, Boglárka Szücs, Márta M-Hamvas, Attila Kiss-Szikszai, György Vámosi, Csaba Máthé

**Affiliations:** ^1^Department of Botany, Faculty of Science and Technology, University of Debrecen, Debrecen, Hungary; ^2^Department of Medical Chemistry, Faculty of Medicine, University of Debrecen, Debrecen, Hungary; ^3^Department of Organic Chemistry, Faculty of Science and Technology, University of Debrecen, Debrecen, Hungary; ^4^Department of Biophysics and Cell Biology, Faculty of Medicine, University of Debrecen, Debrecen, Hungary

**Keywords:** allyl isothiocyanate, microcystin-LR, metaphase block, protein phosphatase type 1 and 2A, phosphohistone H3 Ser10, *Vicia faba*

## Abstract

Horseradish allyl isothiocyanate (AITC, a volatile oil) and cyanobacterial microcystin-LR (MCY-LR, a cyclic heptapeptide) affect eukaryotic cell cycle. MCY-LR inhibits protein phosphatases PP1 and PP2A. We aimed to reveal the mechanisms of their cellular effects in a model eukaryote, *Vicia faba*. We have shown for the first time that AITC had minor effects on PP1 and PP2A activities *in vitro*, but it inhibited significantly PP1 *in vivo*. The combination of 10 μM AITC with 10 μM MCY-LR induced metaphase arrest after short-term (12 h) treatments. 10 μM AITC, 0.2–10 μM MCY-LR and their combinations induced histone H3 hyperphosphorylation, associated with the regulation of metaphase-anaphase transition. This hyperphosphorylation event occurred at any treatment which led to the inhibition of PP1 activity. 10 μM AITC + 10 μM MCY-LR increased the frequency of metaphase spindle anomalies, associated with metaphase arrest. We provide new insights into the mechanisms of metaphase-anaphase transition. Metaphase arrest is induced at the concomitant hyperphosphorylation of histone H3, alteration of metaphase spindle assembly and strong inhibition of PP1 + PP2A activity. Near-complete blocking of metaphase-anaphase transition by rapid protein phosphatase inhibition is shown here for the first time in plants, confirming a crucial role of serine-threonine phosphatases in this checkpoint of cell cycle regulation. Tissue-dependent differences in PP1 and PP2A activities induced by AITC and MCY-LR suggest that mainly regulatory subunits are affected. AITC is a potential tool for the study of protein phosphatase function and regulation. We raise the possibility that one of the biochemical events occurring during AITC release upon wounding is the modulation of protein phosphatase dependent signal transduction pathways during the plant defense response.

## Introduction

Allyl-isothiocyanate (AITC) with a chemical formula of CH_2_ = CH-CH_2_-N = C = S is a main component of the volatile oil of several Brassicaceae species. The main AITC source is the root system of horseradish (*Armoracia* sp.). It is a simple lipophylic molecule, produced from degradation by myrosinase of sinigrin, a glucosinolate after wounding of plant tissues (Zhang, 2010; Nguyen et al., 2013).

There are multiple biochemical and cellular/ physiological effects of isothiocyanates and among them, AITC in eukaryotes. In isothiocyanate producing higher plants, it is thought to play an essential role in the defense against herbivores and pathogens, since it has bactericidal and fungicidal activity (Åsberg et al., 2015). For vertebrates, its effects were observed mainly in mammalian tumor cell lines (see the review of Nguyen et al., 2013) and here we list those related to alterations of cell cycle and cell viability:

(i) Inhibition of the expression of carcinogen-activating enzymes (e.g., cytochrome P450/CYP) in mammalian cells.
(ii) Inducing apoptosis in mammalian tumor cell lines, e.g., by caspase activation or down-regulation of Bcl2 expression.
(iii) Cell cycle arrest at G1/S or G2/M transition by down-regulation of the expression (protein levels) of key cell cycle regulators (Cdk1, Cdc25, cyclin B1) as well as cytoskeletal disruption. Microtubular cytoskeleton was totally disrupted in HT29 (human colorectal cancer) cells by 24 h exposure to 12 μM AITC (Smith et al., 2004). It is worth mentioning here that AITC blocks cell cycle in S phase, thus inhibits entry into mitosis in Arabidopsis (a model plant taxonomically belonging to the same family as horseradish) seedlings treated with AITC vapor. DNA synthesis is not affected, but genes needed for mitosis are down-regulated. Moreover, at treatments with 10–30 mM AITC for at least 36 h, cortical microtubules of epidermal pavement cells were completely disrupted (Åsberg et al., 2015).

Other effects of isothiocyanates include anti-inflammatory and antimicrobial properties.

Even though the permeability of plant cell membranes for AITC is not known, its chemical structure is suitable for penetrating plant membranes and several studies demonstrate that it can be transported easily through bacterial, mammalian, and probably fungal membranes (Purhonen et al., 2008; Zhang, 2010; Bertóti et al., 2016). Moreover, AITC affects plant intracellular events like modulation of glutathione-S-transferase (GST) gene expression (Øverby et al., 2015).

Microcystin-LR (MCY-LR) is a cyclic heptapeptide produced by several cyanobacterial genera/species containing two unusual amino acids: *N*-methyldehydroalanine (Mdha) and the hydrophobic D-amino acid, 3-amino-9-methoxy-2-6,8-trimethyl-10-phenyldeca-4, 6-dienoic acid (Adda). Its amino acid sequence is cyclo (-D-Ala^1^-L-Leu^2^-Derythro-β-methylAsp^3^-L-Arg^4^-Adda^5^-D-isoGlu^6^-*N*-methyldehydro-Ala^7^) (Botes et al., 1985; Chen and Xie, 2016). MCY-LR is a potent inhibitor of type 1 and 2A phosphoserine-threonine specific protein phosphatases (PP1 and PP2A), with IC_50_ values at the subnanomolar range in *in vitro* assays (MacKintosh et al., 1990). The Adda residue enters into the hydrophobic groove of phosphatases near the active site, thereby it blocks irreversibly the binding of phosphorylated serine or threonine residues of substrate proteins (Goldberg et al., 1995). PP1 and PP2A are involved in nearly every aspect of intracellular biochemical processes, including cell cycle regulation, signal transduction etc., in all eukaryotes including plants (Luan, 2003). It is not surprising therefore, that the inhibition of their activity alters mitosis and the organization of subcellular structures involved in cell division (Máthé et al., 2016). Besides protein phosphatase inhibition, MCY-LR has a strong reactive oxygen species (ROS) inducing effect in many eukaryotes, that may also contribute to subcellular structure alterations induced by this compound (Máthé et al., 2016; Pflugmacher, 2016).

As seen above, both AITC and MCY-LR may alter mitotic division in eukaryotes. In a preliminary study, we have observed that crude extracts of horseradish roots arrested *Vicia faba* (broad bean, a model eukaryote for the study of mitotic events, Olszewska et al., 1990; Doležel et al., 1999) meristematic cells in metaphase and induced abnormal mitotic spindle organization, when combined with MCY-LR. Our hypothesis was that a compound from horseradish could alter mitotic activity and this could be related to alterations in protein phosphatase activities. Thus, we aimed to study (i) the cytological effects of the main compound of horseradish volatile oil, AITC alone or in combination with MCY-LR in order to see whether it can be used as a tool for studying the regulation of eukaryotic subcellular events; (ii) to investigate whether PP1 and PP2A are modulated during the subcellular changes detected and to answer the question: is a certain type of phosphatases involved in the regulation of certain mitotic events, e.g., metaphase-anaphase transition and mitotic spindle organization? In this study, we provide novel contributions for the understanding of the mechanisms of metaphase-anaphase transition in plants and in eukaryotes in general.

## Materials and Methods

### Chemicals Used for Treatments

Allyl isothiocyanate (AITC) was purchased from Sigma-Aldrich (St. Louis, MO, United States). Microcystin-LR (MCY-LR) was purified essentially by the method of Kós et al. (1995) as modified by Vasas et al. (2004): *Microcystis aeruginosa* BGSD-243 cells were harvested by centrifugation. The pelleted cells were extracted with 90% methanol. The combined supernatants were dried and dissolved in 5 mM Tris–HCl, pH 7.5. The solution was loaded onto a DEAE (Diethylaminoethyl) (Whatman DE 52) column and flushed with a gradient between 0 and 150 mM NaCl in 5 mM Tris–HCl buffer, pH 7.5. The MCY containing fractions were loaded onto a Toyopearl size exclusion column (80 × 3 cm Toyopearl HW-40) equilibrated with 50% ethanol. The identical fractions were applied to C-18 HPLC column (Supelcosil TM SPLC-18, 25 cm, 10 mm, 5 mm). HPLC analyses were performed with a Shimadzu HPLC system equipped with a Shimadzu SPD-M 10 AVP diode array. The distinctive peaks of the chromatogram were checked by MS (details see below). After rechromatography of the MCY-LR containing fraction, the purified microcystin was free of contaminants.

Okadaic acid (OA) and tautomycin (Tm) used in this study were purchased from Sigma-Aldrich.

### LC-MS

MCY-LR alone or together with AITC were dissolved in sucrose-free modified MS liquid culture medium. Their final concentrations were 10 μM for each. These solutions were incubated at 22°C for 12 h to establish conditions nearly identical to the conditions of plant growth and treatments (see below). This was followed by lyophilization with a Martin-Christ lyophilizer. The dry matter resulted was re-dissolved in 20 μl double distilled water, then 180 μl of ultrapure methanol (VWR, ıRadnor, PA, United States) was added for pelleting of salts. After vortexing and centrifugation (13,000 rpm, 2 min.) with a Heraeus Biofuge, 1 μl of samples were used for further analysis. For LC-MS, a Thermo Accela HPLC was used, with a Thermo LTQ XL Linear Ion Trap Mass Spectrometer as detector. The column was a Phenomenex Kinetex XB-C_18_ column (100 mm × 2.1 mm × 2.6 μm). Oven temperature was maintained at 30°C; the flow rate was 250 μL min^-1^. Eluent A was water with 0.1% formic acid and eluent B was acetonitrile with 0.1% formic acid. The following gradient elution program was used: 0 min, 10% B, 0–10 min, 70% B; 10–11 min, 100% B; 11–16 min, 100% B; 16–18 min, 10% B; 18–20 min, 10% B. One μL of the samples was injected. The mass spectrometer operated in positive ion mode, using the following parameters: source temperature 250°C, capillary temperature 375°C, spray voltage 6.0 kV. The mass range scanned was 300–1500 m/z. Sheath gas and aux flow rates were 10 and 5 arb, respectively. MCY-LR was detected as [M+H]^+^ at m/z 995.5.

### Plant Material and Treatments

*Vicia faba* L. (broad bean/ faba bean, Cv. Lippói) seeds were surface sterilized, then pregerminated to obtain seedlings with young, at least 15 mm long lateral roots ideal for the study of meristematic tissues. At this developmental stage, seedlings were ready for the start of AITC and/or MCY-LR treatments made according to Beyer et al., 2012. In brief, AITC and/or MCY-LR were added to 5 ml of liquid, modified MS medium (MS medium and Gamborg’s vitamins, Murashige and Skoog, 1962; Gamborg et al., 1968) and treatment lasted for 12 h in case of non-synchronized root meristematic cells. OA and Tm stock solutions used in this study were prepared with DMSO, thus for these treatments, control culture medium contained DMSO in an amount equivalent to DMSO content of media containing the inhibitors (their final concentration was 0.2 μM). For synchronization of lateral root meristematic cells, seedlings were treated for 16 h with 2.5 mM hydroxyurea (HU, Sigma-Aldrich) according to Doležel et al. (1999) and mitotic activities were monitored for 24 h after HU washout in the presence of AITC and/or MCY-LR. The tips of lateral roots were used for cytological analyses.

### Immunohistochemistry and Histochemistry

For the labeling of microtubules and chromatin, we fixed lateral root tips with 2% (w/v) paraformaldehyde, then cryosectioned them, used the Cy3-conjugated anti-ß tubulin antibody and 4′,6′-diamidino-2-phenylindole (DAPI) and followed the protocol described before (Máthé et al., 2009). Briefly, sections were washed with PBS, then permeabilized for 10 min with 0.5% (v/v) Triton X-100 (Reanal, Budapest, Hungary). Microtubules were labeled for 8–16 h with the antibody diluted 30-fold in PBS that contained 1% (w/v) bovine serum albumin (Sigma-Aldrich, St. Louis, MO, United States). Sections were washed with PBS and stained for 40 min. with 3 μg ml^-1^ DAPI. Labeling for phosphorylated histone H3 at Ser10 (pH3 Ser10) was performed by indirect immunohistochemistry according to Beyer et al. (2012): treatment with 50-fold diluted rabbit primary anti-p-H3-Ser10 antibody (cat. 05–817, Upstate, Lake Placid, NY, United States) was at 4°C, 12 h, followed by adding of the 100-fold diluted secondary antibody (Alexa 488 conjugated goat anti-rabbit IgG, Molecular Probes, Eugene, OR, United States) at 37°C, 4 h. If microtubules, chromatin and pH3 Ser10 were all labeled in the same root tip section, the sequence of labeling was: primary antibody for pH3 Ser10, Alexa 488- conjugated secondary antibody for pH3 Ser10 + Cy3 conjugated anti-ß tubulin antibody, DAPI. Labeling intensities for pH3 Ser 10 were quantified with the aid of ImageJ64 software: intensities were expressed as area integrated optical density (AIOD).

Mitotic figures were observed both in non-synchronized and synchronized meristems and mitotic indices as well as indices for particular mitotic phases were determined based on chromatin and microtubule labels. Meanwhile, abnormal mitotic figures (both chromatin and mitotic spindle anomalies) were monitored. Abnormal spindle development was given as the percentage of aberrant metaphase spindles out of total metaphase spindle number. Meristematic cells giving rise to vascular tissue and from the region of quiescent center were excluded from observations, the former because cells that will differentiate to tracheary elements are presenting abnormal mitosis *per se*. Sections of at least six root tips per treatment per experiment were analyzed and all experiments were repeated at least four times.

Chromosome squashes following the combined 12 h treatment with 10 μM AITC and 10 μM MCY-LR were prepared according to a classical carmine-acetic acid staining method (Garda et al., 2015). After immersing of lateral roots in 0.1% (w/v) colchicine (Sigma-Aldrich), they were fixed in 45% (v/v) acetic acid (20 min), and hydrolyzed in 1 N HCl at 60°C for 5 min, then stained with 5% (w/v) carmine-acetic acid for 30 min at 60°C. Microscopic examination for chromatin, microtubules, and pH3 Ser10 was performed with the aid of bright-field and fluorescence facilities of an Olympus Provis AX-70 (Olympus, Tokyo, Japan) microscope. To check accuracy of results for pH3 Ser10 labeling, samples were analyzed with an Olympus FluoView 1000 confocal microscope (excitation wavelength-488 nm, emission wavelength range: 500–530 nm; × 60 water-immersion UPLSAPO objective, with a numerical aperture of 1.2, acquisition software: FV-1000).

For monitoring oxidative stress levels in lateral root tissues treated with AITC and/or MCY-LR, we used the fluorescent dye 2′,7′-dichlorofluorescein-diacetate (DCFH-DA) that labels a wide range of ROS and is excitable at 450–480 nm. We used living lateral roots by the modified method of Guo et al. (2008) as described previously (Garda et al., 2016): unfixed lateral roots were stained with 20 μM DCFH-DA for 30 min. After washing with PBS, the Olympus Provis AX-70 fluorescence microscope was used for observations (excitation wavelength: 450–480 nm) and labeling intensities were quantified with ImageJ64 software- intensities were expressed as AIOD. For, ROS monitoring experiments, at least four roots were examined per treatment and experiments were repeated three times.

### The Assay of Protein Phosphatase Activities

In principle, total protein phosphatase (sum of PP1 and PP2A) as well as PP1 and PP2A activities *in vitro* and *in vivo* were measured as described previously (Máthé et al., 2013). For the *in vitro* experiments, we used purified PP1c and PP2Ac (catalytic subunits of PP1 and PP2A from rabbit skeletal muscle, Gergely et al., 1984), respectively, and measured activities in the absence or presence of AITC and/or MCY-LR. These chemicals were preincubated with the purified enzymes in assay buffer, together (both AITC and MCY-LR in the same time) or sequentially for 5 min, then substrate was added and the enzyme activity was measured. Substrate was ^32^P-MLC20 (MLC20 is the turkey gizzard 20 kDa myosin light chain). For *in vivo* measurements, whole roots or root tips were extracted with a buffer of the following composition: 50 mM Tris–HCl (pH 7.5), 0.1 mM EDTA, 0.2 mM EGTA, 0.1% (w/v) DTT, 0.2 mM PMSF (Sigma-Aldrich), 0.5% (v/v) protease inhibitor cocktail (Roche Applied Science, Indianapolis, IN, United States). Protein content of extracts was determined according to Bradford (1976) to perform the assays with equal amounts of proteins. To assay PP1 and PP2A activities separately *in vivo*, Inhibitor-2 (I-2) protein was added at 2 μM (Dedinszki et al., 2015). I-2 is a known natural inhibitor of PP1 in all eukaryotes, including plants (Templeton et al., 2011). Specific protein phosphatase activities were expressed as pmol [^32^Pi] released mg protein^-1^ (Erdődi et al., 1995) and plotted as the percentage of control activities (100%). For *in vivo* experiments, PP2A activity was considered to be the activity of extracts after adding I-2 and PP1 activity was calculated from the difference of total protein phosphatase and PP2A activity.

All protein phosphatase assays were performed as three parallel measurements per treatment and three separate experimental repetitions.

### Data Analysis

All measurements were plotted with the aid of Systat Sigma Plot 10.0^®^ software. The mean ± SE values are represented on the plots. We analyzed statistical significance of treatments vs. controls by Kruskal-Wallis one-way analysis of variance on ranks. All-pairwise significances were analyzed by Dunn’s method together with the Kruskal-Wallis one-way ANOVA, where appropriate.

## Results

AITC is known to bind to the thiol group of cysteine as a free amino acid or in polypeptides (Zhang, 2010). We have predicted that since MCY-LR does not have any sulfur-containing amino acids, it is not likely to form any complex with AITC. Even though during LC-MS analysis, a theoretical dithiocarbamate adduct that is formed on the Arg sidechain in the reaction of MCY-LR with AITC was sought at [M+H]^+^ m/z 1094.5, this theoretical situation occurs only at long-term (several days) of co-existence of AITC and MCY-LR in the same solution. Thus, LC-MS analysis did not reveal any significant reaction product or adduct between AITC and MCY-LR for short-term (12 h) that was used in our experiments (Supplementary Figure S1). As a consequence, the subcellular changes at combined treatments with AITC and MCY-LR detected in the present study are not the effect of a reaction product of these two compounds.

### AITC and MCY-LR Induce Changes in Mitotic Activities and the Organization of Mitotic Structures in *V. faba* Lateral Root Meristems

**FIGURE 1:**
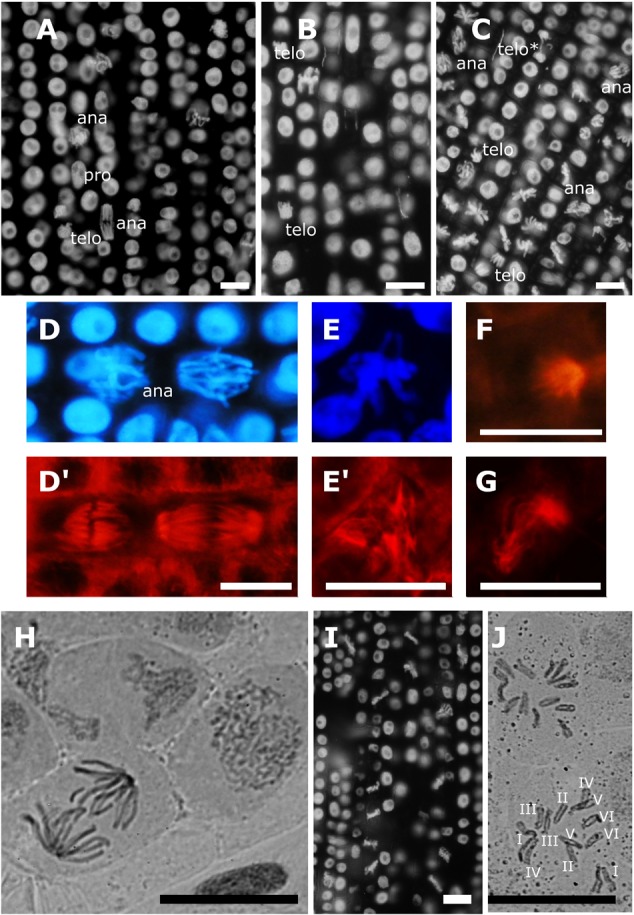
Effects of 12 h treatments with Allyl-isothiocyanate (AITC) and Microcystin-LR (MCY-LR) on chromatin and MT organization. **(A–D,I)** mitotic phases (pro- prophase, ana- anaphase, telo- telophase/cytokinesis) are labeled except metaphases. Chromatin is labeled with 4′,6′-diamidino-2-phenylindole (DAPI) in panels **A–E,I**. MTs are labeled with anti-β-tubulin in panels **D’,E’,F,G**. **(A)** control lateral root tip, no metaphase synchronization is observed; **(B)** treatment with 10 μM AITC; **(C)** treatment with 10 μM MCY-LR, showing the abundance of metaphase cells. (telo^∗^) is an aberrant telophase with a lagging chromosome (see detail on Supplementary Figure S2). Normal chromatin **(D)** and mitotic spindle **(D’)** organization in control cells. **(E–G)** alterations induced by the combined treatment with 10 μM AITC and 10 μM MCY-LR: **(E’)** tripolar spindle with its corresponding metaphase chromatin organization **(E)**; **(F)** monopolar spindle; **(G)** C-shaped spindle. **(H)** control cells, chromosome preparation/ carmine acetate staining. Chromosomes are decondensed, no metaphase synchronization is observed. **(I)** synchronization of cells in metaphase and hypercondensation of chromosomes after combined treatment with 10 μM AITC and 10 μM MCY-LR. **(J)** chromosome preparation/ carmine acetate staining after combined treatment with 10 μM AITC and 10 μM MCY-LR. Typical, distinct *V. faba* chromosomes are seen, 2*n* = 12. Scalebars: 10 μm.

**FIGURE 2:**
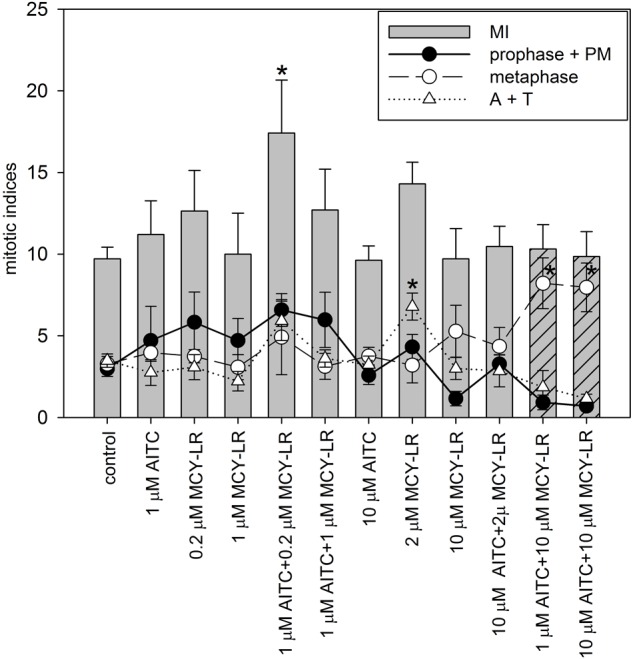
Effects of AITC and MCY-LR on the mitotic activity of non-synchronized *V. faba* lateral root tip meristematic cells. MI in legend is the total mitotic index, PM: prometaphase, A+T: late mitotic (anaphase + telophase) index. ^∗^Significant differences of treatments to controls (*P* < 0.05), *n* ≥ 12. MIs (columns) for the combined treatments of 1 and 10 μM AITC + 10 μM MCY-LR are dashed to highlight their importance among treatments, i.e., non-significant change in MI, but the occurrence of metaphase block.

Observation of chromatin and microtubules in non-synchronized meristematic cells revealed normal mitotic figures in controls and at 10 μM AITC treatments (Figures 1A,B,D,D’). For 10 μM MCY-LR treatments, the abundance of metaphases was characteristic, but all other mitotic phases were regularly observed (Figure 1C). Quantitation of mitotic activities confirmed these observations (Figure 2). Mitotic spindle organization was normal as compared to controls at single chemical treatments (AITC or MCY-LR only) (Supplementary Figure S3). However, 10 μM MCY-LR induced sporadically the appearance of lagging chromosomes in late mitosis (Figure 1C, telo^∗^; Supplementary Figure S2). At combined treatments (10 μM AITC + 10 μM MCY-LR), the 12 h exposure induced a significant increase in the frequency of abnormal metaphase spindles (Supplementary Figure S3). We detected three types of aberrations: tripolar, monopolar, and C-shaped spindles (Figures 1D–G). It is worth mentioning that MCY-LR used alone induced the formation of similar types of abnormal spindles, but only after relatively long-term treatments of *V. faba* meristematic cells (2 and 6 days, Garda et al., 2016). Concomitantly with abnormal spindle organization, in the presence of the 10 μM AITC + 10 μM MCY-LR combination, nearly all mitotic cells were in metaphase (Figures 1I, 2). Chromosome squashes done by a classical method were unsuccessful for controls, because of the high frequency of non-metaphase mitotic cells (Figure 1H). In contrast, chromosome squashes prepared after the combined AITC + MCY-LR treatment revealed well spread, intact chromosomes suitable for karyotype analysis and showed clearly the 2*n* = 2*x* = 12 chromosome number characteristic for *V. faba* (Figure 1J).

Analysis of mitotic indices in meristems of non-synchronized roots at different AITC and MCY-LR concentrations used alone or in combinations revealed the following changes (Figure 2): (i) significant changes of total mitotic activity were induced only at a combination of low AITC (1 μM) and MCY-LR (0.2 μM) concentrations, that had a stimulatory effect; (ii) 2 μM MCY-LR increased significantly the frequency of late mitotic cells, and most importantly (iii) 10 μM MCY-LR and 10 μM AITC + 10 μM MCY-LR increased significantly the frequency of metaphases, and as shown above, the AITC and MCY-LR combination induced metaphase arrest, with very low frequencies of cells in other mitotic phases- 85.04 ± 17.79% of mitotic cells were in metaphase. Moreover, if 10 μM MCY-LR was combined with only 1 μM AITC, similar metaphase arrest occurred, with 79.85 ± 15.11% of mitotic cells being in metaphase (Figure 2).

**FIGURE 3:**
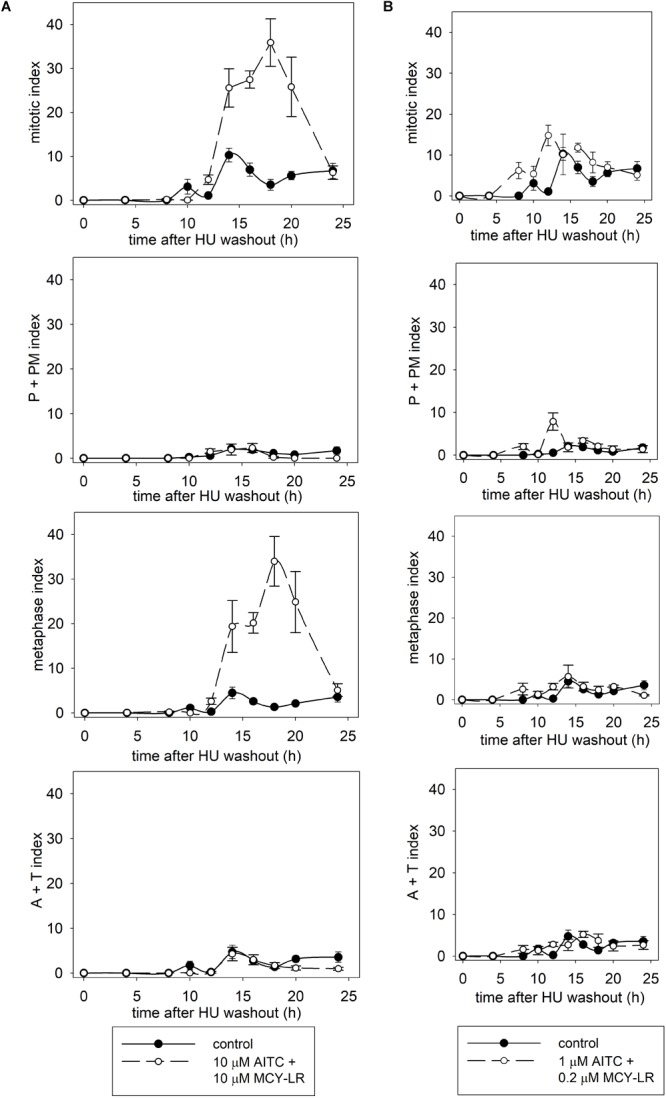
Effects of AITC and MCY-LR on the mitotic activity of HU-synchronized *V. faba* lateral root tip meristematic cells. **(A)** Effects of 10 μM AITC combined with 10 μM MCY-LR. **(B)** Effects of 1 μM AITC combined with 0.2 μM MCY-LR. P+PM index shows the percentage of cells in prophase and prometaphase. A+T (anaphase + telophase) index shows the percentage of cells in late mitosis. *n* ≥ 12.

Synchronization of lateral root tip meristems with HU revealed more detailed information of the mitotic changes induced by AITC + MCY-LR combinations. 10 μM AITC + 10 μM MCY-LR accelerated the onset of mitosis, but blocked cells in metaphase, keeping them at this stage even at 20 h after HU washout, when mitosis was already completed in controls (Figure 3A). 1 μM AITC + 0.2 μM MCY-LR, the combination that stimulated mitosis in non-synchronized meristems (see Figure 2), accelerated the onset of mitosis as well, and although it induced a slight delay of mitotic exit, it confirmed the general mitotic stimulatory effect of this treatment and did not visibly block cells in any mitotic phase. This can be seen particularly for the total mitotic index, which shows that in controls, high mitotic activity is observed at 10–16 h after HU washout, while for treatment duration of high mitotic activity is extended, being at 8–18 h after HU washout (Figure 3B).

### AITC and MCY-LR Do Not Induce Oxidative Stress in *V. faba* Root Tips

The same AITC-MCY-treatments as for monitoring mitotic activities were used for this study. None of the treatments induced significant changes of ROS levels in lateral root tips as compared to controls. The 10 μM AITC + 10 μM MCY-LR combination induced even a slight decrease of ROS (Supplementary Figure S4). This observation confirmed our previous finding that the *V. faba* cultivar used in this study is resistant to oxidative stress, due to efficient non-enzymatic and enzymatic defense systems (Garda et al., 2016). Therefore, we concentrated on the changes in protein phosphatase activities in our further studies.

### AITC and MCY-LR Modulate Protein Phosphatase Activities *in vitro* and *in vivo*

**FIGURE 4:**
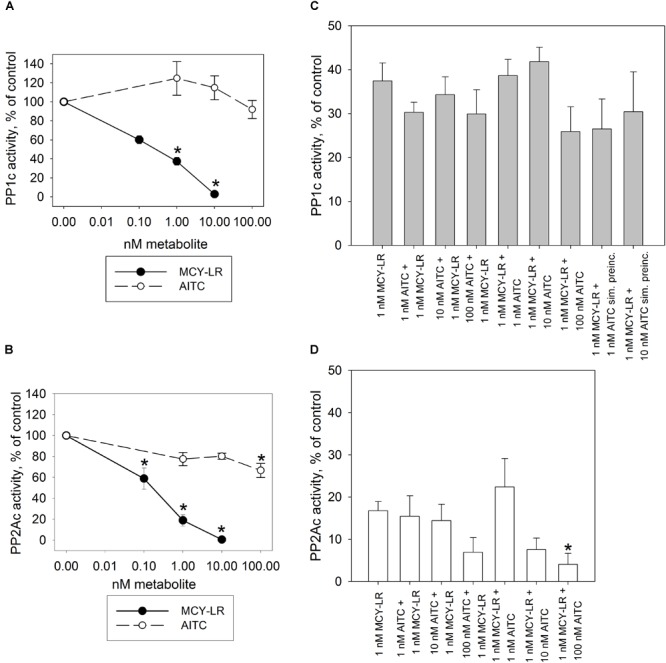
Effects of AITC and MCY-LR on PP1c **(A,C)** and PP2Ac **(B–D)** activities *in vitro*. **(A,B)** Effects of single metabolites in the percentage of control (no treatment) (100%). **(C,D)** Effects of AITC + MCY-LR combinations as compared to the treatment with 1 μM MCY-LR. Activities are expressed here as the percentages of controls (no treatment, 100%) but the statistical significance of the differences between single MCY-LR and combined treatments were analyzed as well. When the combinations of the two compounds were used, preincubation (see Materials and Methods) was started with the compound named firstly. “sim. preinc” means that the two compounds were preincubated simultaneously. PP1c and PP2Ac refers to the catalytic subunits of the protein phosphatases. ^∗^Significant differences of treatments to controls (*P* < 0.05), *n* ≥ 6.

For the *in vitro* assays of PP1c and PP2Ac, MCY-LR had the expected effect: it inhibited their activities with similar potency, with 50% inhibitory concentrations (IC_50_) in the subnanomolar range (Figures 4A,B). Single treatments with AITC did not have significant effects on PP1c and PP2Ac activities, except that 100 nM AITC had a slight, but significant inhibitory effect on PP2Ac (Figure 4B). AITC did not influence significantly the PP1c inhibitory effect of MCY-LR (Figure 4C). AITC amplified the PP2Ac inhibitory effect of MCY-LR, but this effect was significant only when preincubation with 1 nM MCY-LR was followed by preincubation with 100 nM AITC (compare 1 nM MCY-LR with 1 nM MCY-LR + 100 nM AITC on Figure 4D). Even in this case, when we compared different types of combined 100 nM AITC + 1 nM MCY-LR treatments, there were slight differences depending on the sequence of preincubation (MCY-LR, then AITC; AITC, then MCY-LR; simultaneous preincubation), but these differences proved to be non-significant.

**FIGURE 5:**
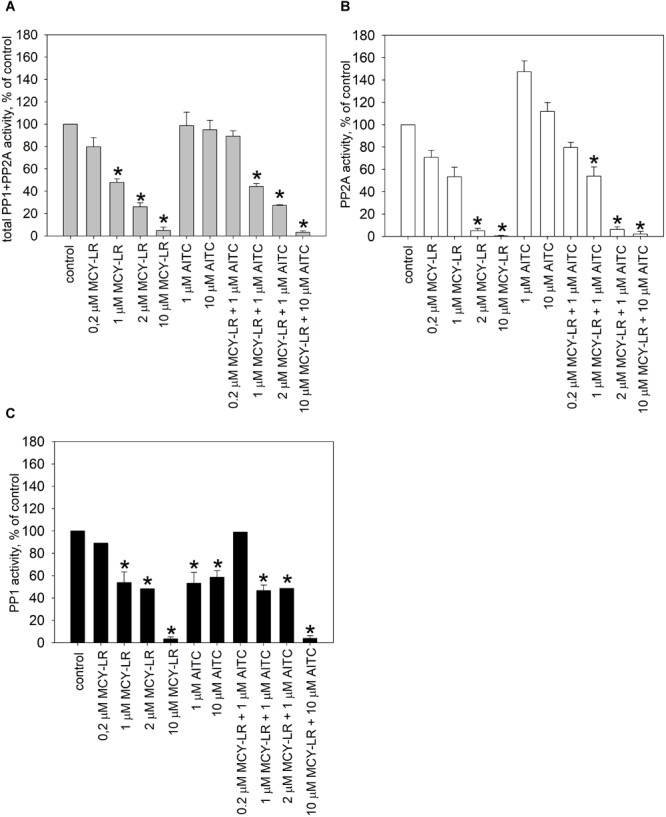
Effects of AITC and MCY-LR on total protein phosphatase (PP1+PP2A) **(A)**, PP2A **(B)**, and PP1 **(C)** activities of *V. faba* whole lateral roots *in vivo*. ^∗^Significant differences of treatments to controls (*P* < 0.05), *n* ≥ 6.

**FIGURE 6:**
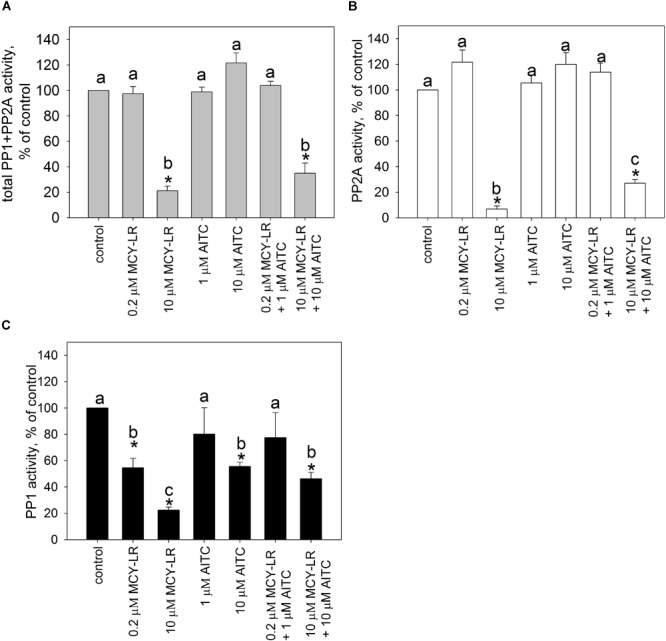
Effects of AITC and MCY-LR on total protein phosphatase (PP1+PP2A) **(A)**, PP2A **(B)**, and PP1 **(C)** activities of *V. faba* tips of lateral roots *in vivo*. ^∗^Significant differences of treatments to controls (*P* < 0.05), *n* ≥ 6. All-pairwise multiple comparisons are indicated by letter codes.

For the *in vivo* protein phosphatase assays, one should take into consideration that for the total pool of serine-threonine protein phosphatases in a eukaryotic cell, PP2A activity is predominant, although PP1 activity has crucial roles as well (Shi, 2009; Virshup and Shenolikar, 2009). Our preliminary studies confirmed this in *V. faba* roots. For the tips of lateral roots, the main subject of our study, PP2A activity was 63.48 ± 4.3% of the total PP1+PP2A activity in controls. We measured activities both in extracts of whole lateral roots and root tips treated for 12 h and found important similarities and differences for these two extract types (Figures 5, 6). In case of whole lateral roots, MCY-LR induced a dose-dependent inhibition of total PP1 + PP2A activities, and the IC_50_ was around 1 μM MCY-LR for PP1 and PP2A, respectively (Figures 5A–C). MCY-LR had a higher inhibitory effect on PP2A, than on PP1: at 2 μM, PP2A activity was 5.12 ± 2.16 (SE)%, while PP1 activity was 48.27 ± 0.2 (SE)% of controls (Figures 5B,C). Single treatments with AITC did not induce significant changes of total phosphatase activity. Even though apparently, 1 μM AITC stimulated PP2A, this stimulation was not statistically significant (Figure 5B). On the other hand, 1, and 10 μM AITC inhibited significantly PP1 activity (Figure 5C). When AITC was combined with MCY-LR, it did not influence significantly the PP1 or PP2A activities of MCY-LR (Figures 5B,C). Differences in case of root tips (as compared to whole lateral roots) were as follows (Figure 6): (i) 0.2 μM MCY-LR inhibited PP1 in a significant manner (to near IC_50_ value), but it had a slight stimulatory effect on PP2A. 10 μM MCY-LR inhibited PP2A more potently, than PP1, indicating that in general, this inhibitor has higher potency for PP2A in *V. faba*; (ii) AITC had a significant inhibitory effect on PP1 only at 10 μM; (iii) the combined treatment of 10 μM AITC + 10 μM MCY-LR inhibited PP1 and PP2A activities to a significant, but lesser degree, than MCY-LR alone. These differences between the single 10 μM MCY-LR and the combined treatments proved to be significant (Figures 6B,C).

Two other protein phosphatase inhibitors were assayed for their protein phosphatase inhibitory effects in *V. faba* lateral root tips. Both OA and Tm inhibited the total PP1 + PP2A activity (Supplementary Figure S5A). PP2A was inhibited significantly only by OA, while PP1 activity was inhibited significantly only by Tm (Supplementary Figures S5B,C).

### AITC and MCY-LR Alone or in Combinations Induce Histone H3 Hyperphosphorylation at Ser10

**FIGURE 7:**
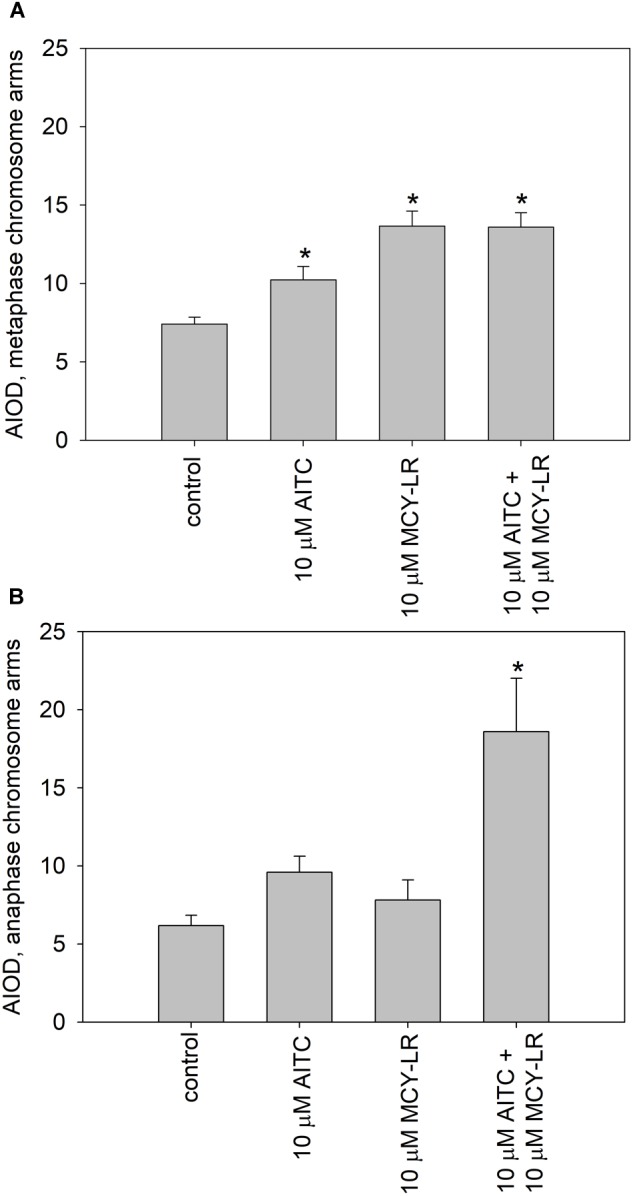
Effects of high AITC and MCY-LR concentrations on histone H3 Ser10 phosphorylation in metaphase **(A)** and anaphase **(B)** cells. ^∗^Significant differences of treatments to controls (*P* < 0.05), *n* ≥ 13.

We have labeled pH3 Ser10 in meristematic cells and analyzed the degree of histone H3 phosphorylation in metaphase cells. Control metaphase cells showed normal pH3 Ser10 labeling patterns for plants as shown before (Schroeder-Reiter et al., 2003; Beyer et al., 2012). That is, intense labeling was characteristic for the pericentromeric region of chromosomes, while chromosome arms showed weaker pH3 Ser10 signal (Supplementary Figure S6A). Treatments with 10 μM AITC, 10 μM MCY-LR as well as the combination of 10 μM AITC + 10 μM MCY-LR maintained the intense labeling of pericentromeric regions and increased labeling intensity in metaphase chromosome arms. For the combined treatment, this was accompanied by visible hypercondensation of chromosomes (Supplementary Figures S6B–D). Quantification of labeling intensities in chromosome arms revealed that the above changes were significant for all treatments (Figure 7A). At lower concentrations of AITC and MCY-LR, we observed that only 0.2 μM MCY-LR induced a significant increase of labeling intensity in chromosome arms (Supplementary Figure S7A).

Control anaphase cells showed maintenance of intense labeling of pericentromeric zones, but weaker pH3 Ser10 signal in chromosome arms, than metaphase chromosomes, as expected (Manzanero et al., 2002; Houben et al., 2007; Supplementary Figure S6E). As for metaphase cells, treatments with 10 μM AITC, 10 μM MCY-LR as well as the combination of 10 μM AITC + 10 μM MCY-LR maintained the intense labeling of pericentromeric regions and increased labeling intensity in chromosome arms. This increase was prominent for the combined treatment (Supplementary Figures S6F–H). Quantification of labeling intensities in anaphase chromosome arms revealed that the above changes were well visible for the treatment with 10 μM AITC and statistically significant for the combined treatment, while 10 μM MCY-LR induced only a slight increase (Figure 7B). At lower concentrations of AITC and MCY-LR, changes in the degree of histone H3 phosphorylation were non-significant. 1 μM AITC and 0.2 μM MCY-LR used separately induced slight increases, while the combined treatment induced a slight decrease in the intensity of pH3 Ser 10 labels (Supplementary Figure S7B). OA and Tm increased the phosphorylation of histone H3, but this was significant only for Tm for metaphase cells (Supplementary Figure S7C). For anaphase cells, no significant changes were observed at treatments with these two inhibitors (Supplementary Figure S7D).

## Discussion

The most important cytological effects of AITC alone or in combination with MCY-LR can be summarized as follows: (i) hyperphosphorylation of histone H3 at Ser10 in metaphase and anaphase chromosome arms; (ii) the mitotic stimulatory effects of low concentration AITC and MCY-LR combinations, and most importantly, (iii) combinations of high AITC and MCY-LR concentrations induced not only histone H3 hyperphosphorylation, but abnormal mitotic spindle organization and metaphase arrest as well. Metaphase arrest was observed both in synchronized and non-synchronized cells, and was comparable to the effects of colchicine (Pan et al., 1993) and the combined treatment was suitable for high quality chromosome preparation for karyotyping (see Figures 1–3A, 7 and Supplementary Figures S3, S6, S7).

Both AITC and MCY-LR are known for inducing oxidative stress in all eukaryotes including plants, although for AITC this was described only at very high doses (Nguyen et al., 2013; Máthé et al., 2016; Pflugmacher, 2016). However, we proved that in *V. faba* roots, none of these compounds increases ROS levels (Supplementary Figure S4). *V. faba* has been proven before to be resistant to oxidative stress, since H_2_O_2_ and MCY-LR did not increase ROS levels, but increased the activity of antioxidant enzymes (Garda et al., 2016). Since both compounds had significant effects on PP1 and PP2A activities *in vivo* and/or *in vitro*, we will discuss their cytological effects in this context.

It was previously known that both PP1 and PP2A are involved in the dephosphorylation of histone H3 at diverse serine and threonine residues in yeast and animal cells and the inhibition of their activities by diverse drugs will increase the overall level of histone H3 phosphorylation or its hyperphosphorylation at specific residues like Ser10 (Guo et al., 1995; Hsu et al., 2000; Manzanero et al., 2002). Similar events were suggested for plant cells as well (Manzanero et al., 2002; Beyer et al., 2012). For eukaryotic cells other than plants, it is known that Aurora B kinase phosphorylates histone H3, leading to the increase of chromosome condensation. PP1 has a contrary effect, since it dephosphorylates Aurora B substrates involved in chromosome condensation and cohesion, allowing metaphase-anaphase transition (Afonso et al., 2017). Thus, PP1 has an important, if not primary, role in histone H3 dephosphorylation. In animal and human cells, PP2A has also a role in pH3 Ser10 dephosphorylation (Li et al., 1996). For plants, an interaction between PP2A and histone H3 is likely to occur (Bíró et al., 2012), but to date, little was known on the role of PP1 in histone H3 dephosphorylation. In this study, we have observed that AITC used alone or in combinations with MCY-LR induced pH3 Ser10 hyperphosphorylation both in metaphase and anaphase chromosomes at concentrations that inhibited PP1 activity in lateral root tips. This was true even for treatments where PP1 was inhibited, but PP2A activities were not changed significantly *in vivo* (Table 1). For metaphase chromosomes, the increase in the pool of Ser10 phosphorylated histone H3 occurred at 0.2 and 10 μM MCY-LR used in the absence of AITC as well (Figures 6, 7, Supplementary Figure 6, and Table 1). Moreover, OA known to inhibit primarily PP2A (for *in vivo* assays, proven in this study as well, Supplementary Figure S5B), did not induce significant changes in the phosphorylation state of histone H3, while Tm (known to inhibit primarily PP1 activity and proven *in vivo* in this study as well, Supplementary Figure S5C) did induce a significant increase in the phosphorylation of histone H3 in metaphase chromosome arms (Supplementary Figure S7C and Table 1). These data suggest that although the role of PP2A in histone dephosphorylation in plants cannot be ruled out, PP1 seems to play a crucial role in this process.

**Table 1:** A summary of cytological and biochemical effects of 12 h AITC treatments with or without MCY-LR as well as of OA and Tm in lateral root tips.

Treatment	pH3 Ser10 hyperphosphorylation, metaphase/ anaphase	Abnormal spindle development	PP1 inhibition	PP2A inhibition	concomitant PP1 + PP2A inhibition	Metaphase arrest
1 μM AITC	± /-	–	–	–	–	–
10 μM AITC	+/+	–	+	–	–	–
0.2 μM MCY-LR	+/-	–	+	–	–	–
10 μM MCY-LR	+/ ±	–	+	+	+	–
0.2 μM OA	–/-	–	–	+	–	–
0.2 μM Tm	+/-	–	+	–	–	–
1 μM AITC + 0.2 μM MCY-LR	–/-	–	–	–	–	–
10 μM AITC + 10 μM MCY-LR	+/+	+	+	+	+	+

Histone H3 hyperphosphorylation contributes to hypercondensation of metaphase chromosomes and the delay of metaphase-anaphase transition (Manzanero et al., 2002; Beyer et al., 2012). However, in our study total metaphase arrest occurred only at (1)10 μM AITC + 10 μM MCY-LR treatments. Other AITC (with or without MCY-LR) treatments did induce histone H3 hyperphosphorylation without total metaphase arrest (Table 1). We drew the conclusion that hyperphosphorylation of histone H3 at Ser10 by itself is not enough for arrest, other factors must contribute to induce this blocking of metaphase-anaphase transition (see below).

For the induction of abnormal spindle development, this could not be observed in cases when one of the protein phosphatases (PP1 or PP2A) was not inhibited *in vivo* (Table 1). Inhibition of both PP1 and PP2A is needed for the development of such anomalies. In general, this seems to be true not only for the combination of 10 μM AITC and 10 μM MCY-LR, but for long-term treatment with 10 μM MCY-LR applied as the sole inhibitor as well. The latter treatment inhibits both phosphatases, but induces only a slight increase of abnormal spindle frequency after 12 h treatment (Table 1 and Supplementary Figure S3). However, 48 h treatments with 1 and 10 μM MCY-LR increased significantly the formation of abnormal metaphase spindles and induced abnormal sister chromatid segregation, and this could be correlated with inhibition of total PP1 + PP2A activities (Garda et al., 2016). Moreover, long-term (6 days) of MCY-LR (as single inhibitor) treatment, that induces severe protein phosphatase inhibition, high frequency of spindle anomalies and metaphase histone H3 hyperphosphorylation with chromosome hypercondensation- leads to severe inhibition of metaphase/anaphase transition in non-synchronized *V. faba* cells (Beyer et al., 2012; Garda et al., 2016). Abnormal chromatid segregation like the appearance of lagging chromosomes may be induced by MCY-LR solely by histone H3 hyperphosphorylation (without abnormal spindle organization) as shown in this study (Figure 1c and Supplementary Figure S2) and in our previous work (Beyer et al., 2012). Even though treatments with MCY-LR as the sole inhibitor induced such anomalies, the short-term treatments with 1 μM or 10 μM AITC + 10 μM MCY-LR were the only that induced nearly complete metaphase arrest.

To sum up, out of the treatments tested (1 μM AITC + 10 μM MCY-LR treatment was not checked for microtubule organization) only a combination of 10 μM AITC and 10 μM MCY-LR will lead to a substantial increase of the frequency of metaphase spindle anomalies at short-term (12 h) treatments (Figure 1 and Supplementary Figure S3). In this study, the inhibition of both PP1 and PP2A activities induced the development of abnormal spindles (Table 1). This confirms previous studies showing that both PP1 and PP2A are required for normal spindle assembly (Axton et al., 1990; Ayaydin et al., 2000). Thus, metaphase arrest was induced, when the following events occurred concomitantly in root tip meristems (i) histone H3 hyperphosphorylation; (ii) abnormal spindle development; (iii) severe inhibition of both PP1 and PP2A activities (Table 1).

There are various effects of protein phosphatases (and among them, PP1) on metaphase-anaphase transition in eukaryotes. Inhibition of PP1 by neutralizing antibodies leads to metaphase arrest in mammalian cells, but this report did not provide information on alterations of spindle organization or histone H3 phosphorylation (Fernandez et al., 1992). Protein phosphatases affect the functioning of the APC/C complex and their inhibition may lead to the delay of metaphase/anaphase transition in *Xenopus* (Tunquist and Maller, 2003). In contrast, Pinsky et al. (2009) reported that PP1 activity prevents spindle checkpoint activation in budding yeast. In plants, knowledge on the effects of PP1 on the onset of late mitosis is limited. For tobacco BY-2 cells, OA induces prophase arrest, however, this drug inhibits mainly PP2A, not PP1 (Zhang et al., 1992). As a consequence, the present report might give an important contribution to the understanding of the role of PP1 in metaphase-anaphase transition, given the significant histone H3 hyperphosphorylation induced by its inhibition.

In the following sections, we attempt to explain the effects of AITC treatments (with or without MCY-LR) on protein phosphatase activities, mainly *in vivo*.

MCY-LR has been known for a long time as a potent inhibitor of PP1 and PP2A and it also inhibits a number of other protein phosphatases of the PPP family-PP4, PP5, PP6 (MacKintosh et al., 1990; Fontanillo and Köhn, 2018). It should be noted that there is a controversy in literature regarding the preference of this heptapeptide to a given type of phosphatase. While several authors state that it inhibits PP1 and PP2A with equal potency (MacKintosh and Diplexcito, 2003), others claim that it has a preference to PP2A over PP1 (Honkanen et al., 1990, 1994). Here we demonstrate that in the *in vitro* assay for the activity of catalytic subunits, the inhibition of PP1 and PP2A by microcystin is similar (Figure 4). In contrast, for *V.faba* root extracts, the inhibitory effect is tissue-specific, but in principle PP2A is preferentially inhibited (Figures 5, 6). However, it should be noted that the *in vitro* study is not in accordance with the early finding of Honkanen et al. (1990), who found that even during the *in vitro* assay with purified PP1 or PP2A, MCY-LR inhibits PP2A more potently than PP1. Thus, the degree of phosphatase inhibition induced by MCY-LR depends on the assay conditions as well. The discrepancy between *in vitro* and *in vivo* assays may be caused by the differences in the nature and activity of protein phosphatase regulatory subunits in different tissues (see below).

As concerning the effects of AITC on protein phosphatase activities *in vivo*, one should take into consideration, that even though it is an unstable compound, it is probable that at least a fraction remains intact after entering cells during the 12 h exposure period used in this study. On the other hand it is known that a given fraction of compounds of horseradish essential oil can be detoxified by GST in *Candida albicans* (Bertóti et al., 2016) and this was described for pure AITC in mammals (Zhang, 2010). AITC increases GST gene expression in the model plant Arabidopsis (Øverby et al., 2015). Thus, it is likely that intracellular AITC is partly detoxified by eukaryotic cells. If we admit that the remaining free AITC interacts directly with PP1/PP2A *in vivo*, the chemical structure of this isothiocyanate makes highly probable its ability to exert nucleophilic attack against several amino acids including cysteine (e.g., the probable mechanism of its conjugation to glutathione). Indeed, AITC interacts with cysteine residues of proteins and this is probably a covalent interaction (Zhang, 2010). An early study showed that PP1 and PP2A activity is inhibited by reagents that modify sulfhydryl groups (Nemani and Lee, 1993). Later on, Choi et al. (2017) proved that binding of tautomycetin to Cys127 of PP1 is the key mechanism of its inhibition of PP1 activity. This reactive Cys residue is also involved in the inhibition of PP1 by a small inorganic molecule: H_2_S, which induces persulfidation (Yadav et al., 2017). MCY-LR binds covalently to Cys273 of PP1c, also important for PP1 inhibition (Fontanillo and Köhn, 2018). Even if we accept a possible binding to cysteine residues, AITC does not affect significantly PP1c activity *in vitro* (Figure 4). This means that AITC probably does not bind to Cys273. The PP1 inhibitory (that is, opposite) effect of 10 μM AITC *in vivo* might be related to its complex interaction with regulatory and catalytic subunits (see below). Concerning PP2A activities, drugs like phoslactomycin A that bind to the Cys269 residue of PP2Ac important for its activity are inhibiting PP2A (Teruya et al., 2005). A mutation characterized by the change of this Cys residue to Gly leads to OA resistance (Shima et al., 1994). Interestingly, MCY-LR also binds to Cys269 of PP2A by its Mdha residue, and although the Adda residue is responsible mainly for PP1 and PP2A inhibition, the Cys binding also influences the effect of MCY-LR on PP2A activity (Pereira et al., 2011; Fontanillo and Köhn, 2018). AITC is not likely to interact with Cys269, because it does not produce adducts with MCY-LR, but preincubation with 1 nM MCY-LR firstly, then with 100 nM AITC induces more severe inhibition of PP2A, than MCY-LR alone (see Figure 4D and Supplementary Figure S1). To sum up our *in vitro* protein phosphatase inhibition assays, at least for PP1, AITC does not interact significantly with the catalytic subunit (Figure 4).

On the other hand, we have two reasons to presume that *in vivo*, AITC interacts with regulatory subunits of PP1 and PP2A: (i) the differences between *in vitro* and *in vivo* AITC effects on protein phosphatase activities: no significant effects (alone or in combinations with MCY-LR) on PP1c activities *in vitro* and the significant inhibition of PP1 activities by AITC both in whole roots and root tips *in vivo* (compare Figures 4–6); (ii) the tissue specific modulation of protein phosphatase activities by AITC (and MCY-LR) (see Figures 5, 6). Moreover, even though both 10 μM MCY-LR and the 10 μM AITC + 10 μM MCY-LR combination induce severe PP1 and PP2A inhibition *in vivo*, the degree of inhibition at the combined treatment is significantly lower, than for the single-MCY-LR treatment (Figures 6B,C), but this is valid for root tips and not for whole root extracts (Figures 5B,C). In the next section, we present the second argument in detail.

For the *in vivo* protein phosphatase assays, AITC modulates their activities in a tissue-specific manner. 1 μM AITC inhibits only PP1 in whole lateral roots, but its effects are non-significant in lateral root tips where the meristematic tissue- subject of our study- occurs (compare Figures 5, 6). In eukaryotes, PP1 and PP2A are in fact complexes containing catalytic as well as a various number of regulatory subunits. Regulatory subunits are responsible for the diversity and tissue-specificity of functions of the catalytic subunits (Shi, 2009). This is valid for plants, too, where, as in other multicellular organisms, regulation of PP1 activity is diverse in different tissues and/or subcellular compartments (for example, RSS1 is involved in the regulation of cell cycle by this phosphatase). An interesting example is Inhibitor 3 (INH3), a regulator of PP1 found in *V. faba* and Arabidopsis. It is expressed in diverse organs and tissues, with particularly strong expression in Arabidopsis root tips (Takemiya et al., 2009). Diverse PP1 regulatory subunits were proposed for contributing to control of the spindle assembly checkpoint in eukaryotes (Bollen et al., 2009). Regulatory subunits of PP2A are even more diverse, than for PP1, being classified into classes B, B’, B,” and B”’ (Shi, 2009; Uhrig et al., 2013). The *Ton2/Fass* gene family encodes for a B” regulatory subunit, involved in the regulation of the onset of mitosis and the assembly of mitotic microtubules (Spinner et al., 2013).

To sum up the biochemical mechanisms of AITC-protein phosphatase interaction(s), at present we do not have enough data to construct a model for this interaction. However, as shown above, AITC may also affect the interaction of core enzymes with or act directly on their regulatory subunits in a plant tissue-specific manner. An important future task is to reveal these molecular interactions. The effects of AITC on PP1 and PP2A activities raise the possibility that this volatile oil, alone or in combination with naturally occurring plant protein phosphatase inhibitors (like INH3-like proteins mentioned above) may modulate phosphatase mediated signal transduction pathways involved in defense responses upon wounding in glucosinolate-producing plants.

The main conclusions of the present study are (i) AITC alone or in combination with MCY-LR influences protein phosphatase PP1 and PP2A activities *in vivo*, but generally, does not have significant influences on the catalytic subunits *in vitro*; (ii) we provide experimental evidence that PP1 is a protein phosphatase of crucial importance in pH3 Ser10 dephosphorylation in *V. faba* and (iii) together with the proper organization of spindle assembly, that is dependent on both PP1 and PP2A activities, PP1-modulated histone H3 phosphorylation regulates metaphase-anaphase transition. Concomitant and rapid alterations of these two cellular processes lead to complete metaphase arrest. We have proved that the combinations of AITC with MCY-LR are useful for the more detailed elucidation of protein phosphatase mediated regulation of mitosis in plant cells. It is a future task to investigate whether similar effects could be induced in other eukaryotic, like mammalian cells and to identify the molecular events/ signaling pathways that connect histone hyperphosphorylation, altered spindle organization and protein phosphatase activities to the arrest of cells in metaphase. This report shows for the first time that AITC modulates serine-threonine protein phosphatase activities and that acute PP1 (and PP2A) inhibition induces metaphase arrest in plants.

## Data Availability Statement

The raw data supporting the conclusions of this manuscript will be made available by the authors, without undue reservation, to any qualified researcher.

## Author Contributions

TG performed a significant part of experiments including preparation of extracts for *in vivo* protein phosphatase assays and contributed to data analysis. ZK and FE performed protein phosphatase assays and contributed to data analysis related to these measurements. CF and BS contributed essentially to immunohistochemistry and histochemistry experiments. SG and AK-S performed the LC-MS measurements. GáV purified MCY-LR. MM-H made the statistical analysis. GyV made essential contributions to confocal microscopy observations. CM made microscopical observations, designed experiments, and wrote the manuscript.

## Conflict of Interest Statement

The authors declare that the research was conducted in the absence of any commercial or financial relationships that could be construed as a potential conflict of interest.

## Supplementary Material

FIGURE S1 LC-MS spectrogram of a desalted, sucrose-free liquid MS medium containing 10 μM MCY-LR (red peak) and 10 μM AITC + μM MCY-LR (blue peak) shows no significant difference between the mass spectra.

FIGURE S2 Telophase chromatin from a lateral root tip meristem treated with 10 μM MCY-LR, showing a lagging chromosome (arrow). Scalebar: 10 μm.

FIGURE S3 Effects of 10 μM AITC, 10 μM MCY-LR and their combination on the development of abnormal metaphase spindles: percentage of abnormal spindles out of total metaphase spindles at different metabolite treatments. ^∗^Significant differences of treatments to controls (*P* < 0.05), *n* ≥ 12.

FIGURE S4 Effects of AITC and MCY-LR on the accumulation of reactive oxygen species (ROS) in *V. faba* lateral root tips. ROS was detected by DCFH-DA staining. *n* ≥ 12.

FIGURE S5 Effects of okadaic acid (OA) and Tm on the protein phosphatase activities of lateral roots tips. **(A)** total PP1 + PP2A, **(B)** PP2A, **(C)** PP1. ^∗^Significant differences of treatments to controls (*P* < 0.05), *n* ≥ 6.

FIGURE S6 Conventional fluorescence microscopy images for phospho-histone H3 Ser10 (pH3 Ser10) labeling of metaphase **(A–D)** and anaphase **(E–H)** cells: **(A,E)** controls; treatments with: **(B,F)** 10 μM AITC, **(C,G)** 10 μM MCY-LR, **(D,H)** 10 μM AITC + 10 μM MCY-LR. Scalebars: 10 μm.

FIGURE S7 Effects of low AITC and MCY-LR concentrations on histone H3 Ser10 phosphorylation in metaphase **(A)** and anaphase **(B)** cells. Effects of 0.2 μM OA and 0.2 μM Tm on histone H3 Ser10 phosphorylation in metaphase **(C)** and anaphase **(D)** cells. ^∗^Significant differences of treatments to controls (*P* < 0.05), *n* ≥ 12.

## References

[B1] Afonso O. Figueiredo A. C. Maiato H. (2017). Late mitotic functions of aurora kinases. *Chromosoma* 126 93–103. 10.1007/s00412-016-0594-5 27106516

[B2] Åsberg S. E. Bones A. M. Øverby A. (2015). Allyl isothiocyanate affects the cell cycle of *Arabidopsis thaliana*. *Front. Plant Sci.* 6:364. 10.3389/fpls.2015.00364 26042144 PMC4436579

[B3] Axton J. M. Dombrádi V. Cohen P. T. W. Glover D. M. (1990). One of the protein phosphatase 1 isoenzymes in *Drosophila* is essential for mitosis. *Cell* 63 33–46. 10.1016/0092-8674(90)90286-N 2170019

[B4] Ayaydin F. Vissi E. Mészáros T. Miskolczi P. Kovács I. Fehér A. (2000). Inhibition of serine/threonine-specific protein phosphatases causes premature activation of cdc2MsF kinase at G2/M transition and early microtubule organisation in alfalfa. *Plant J.* 23 85–96. 10.1046/j.1365-313x.2000.00798.x 10929104

[B5] Bertóti R. Vasas G. Gonda S. Nguyen N. M. Szőke É Jakab Á (2016). Glutathione protects *Candida albicans* against horseradish volatile oil. *J. Basic Microbiol.* 56 1–9. 10.1002/jobm.201600082 27272511

[B6] Beyer D. Tándor I. Kónya Z. Bátori R. Roszik J. Vereb G. (2012). Microcystin-LR, a protein phosphatase inhibitor induces alterations in mitotic chromatin and microtubule organization leading to the formation of micronuclei in Vicia faba. *Ann. Bot.* 110 797–808. 10.1093/aob/mcs154 22819947 PMC3423812

[B7] Bíró J. Farkas I. Domoki M. Ötvös K. Bottka S. Dombrádi V. (2012). The histone phosphatase inhibitory property of plant nucleosome assembly protein-related proteins (NRPs). *Plant Physiol. Biochem.* 52 162–168. 10.1016/j.plaphy.2011.12.010 22285370

[B8] Bollen M. Gerlich D. W. Lesage B. (2009). Mitotic phosphatases: from entry guards to exit guides. *Trends Cell Biol.* 19 531–541. 10.1016/j.t.c.b.2009.06.005 19734049

[B9] Botes D. P. Wessels P. L. Kruger H. Runnegar M. T. C. Santikarn S. Smith R. J. (1985). Structural studies on cyanoginosins-LR, YR, YA, and YM, peptide toxins from *Microcystis aeruginosa*. *J. Chem. Soc*. *Perkin Trans.* 1 2747–2748. 10.1039/P19850002747

[B10] Bradford M. M. (1976). A rapid and sensitive method for the quantitation of microgram quantities of protein utilizing the principle of protein-dye binding. *Anal. Biochem.* 72 248–254. 10.1016/0003-2697(76)90527-3 942051

[B11] Chen L. Xie P. (2016). Mechanisms of microcystin-induced cytotoxicity and apoptosis. *Mini Rev. Med. Chem.* 16 1018–1031. 10.2174/1389557516666160219130407 26891929

[B12] Choi M. S. Swingle M. D’Arcy B. Abney K. Rusin S. F. Kettenbach A. N. (2017). PP1:tautomycetin complex reveals a path toward the development of PP1-specific inhibitors. *J. Am. Chem. Soc.* 139 11703–11706. 10.1021/jacs.7b09368 29156132 PMC5729109

[B13] Dedinszki D. Sipos A. Kiss A. Bátori R. Kónya Z. Virág L. (2015). Protein phosphatase-1 is involved in the maintenance of normal homeostasis and in UVA irradiation-induced pathological alterations in HaCaT cells and in mouse skin. *Biochim. Biophys. Acta Mol. Basis Dis.* 1852 22–33. 10.1016/j.bbadis.2014.11.005 25446992

[B14] Doležel J. Číhaliková J. Weiserová J. Lucretti S. (1999). Cell cycle synchronization in plant root meristems. *Meth. Cell Sci.* 21 95–107. 10.1023/A:1009876621187 10728642

[B15] Erdődi F. Tóth B. Hirano K. Hirano M. Hartshorne D. J. Gergely P. (1995). Endothall thioanhydride inhibits protein phosphatases-1 and -2A in vivo. *Am. J. Physiol.* 269 C1176–C1184. 10.1152/ajpcell.1995.269.5.C1176 7491907

[B16] Fernandez A. Brautigan D. L. Lamb N. J. C. (1992). Protein phosphatase type 1 in mammalian cell mitosis: chromosomal localization and involvement in mitotic exit. *J. Cell Biol.* 116 1421–1430. 10.1083/jcb.116.6.1421 1311712 PMC2289383

[B17] Fontanillo M. Köhn M. (2018). Microcystins: synthesis and structure-activity relationship studies towards PP1 and PP2A. *Bioorg. Med. Chem.* 26 1118–1126. 10.1016/j.bmc.2017.08.040 28893598

[B18] Gamborg O. L. Miller R. A. Ojima K. (1968). Nutrient requirements of suspension cultures of soybean root cells. *Exp. Cell Res.* 50 151–158. 10.1016/0014-4827(68)90403-5 5650857

[B19] Garda T. Kónya Z. Tándor I. Beyer D. Vasas G. Erdődi F. (2016). Microcystin-LR induces mitotic spindle assembly disorders in vicia faba by protein phosphatase inhibition and not reactive oxygen species induction. *J. Plant Physiol.* 19 1–11. 10.1016/j.jplph.2016.04.009 27186862

[B20] Garda T. Riba M. Vasas G. Beyer D. M-Hamvas M. Hajdu G. (2015). Cytotoxic effects of cylindrospermopsin in mitotic and non-mitotic vicia faba cells. *Chemosphere* 120 145–153. 10.1016/j.chemosphere.2014.06.035 25016338

[B21] Gergely P. Erdődi F. Bot G. (1984). Heparin inhibits the activity of protein phosphatase-1. *FEBS Lett.* 169 45–48. 10.1016/0014-5793(84)80286-0 6325237

[B22] Goldberg J. Huang H. Kwon Y. Greengard P. Nairn A. C. Kuriyan J. (1995). Three dimensional structure of the catalytic subunit of protein serine/threonine phosphatase-1. *Nature* 376 745–753. 10.1038/376745a0 7651533

[B23] Guo K. Xia K. Yang Z. M. (2008). Regulation of tomato lateral root development by carbon monoxide and involvement in auxin and nitric oxide. *J. Exp. Bot.* 59 3443–3452. 10.1093/jxb/ern194 18653694 PMC2529230

[B24] Guo X. W. Th’ng J. P. H. Swank R. A. Anderson H. J. Tudan C. Bradbury E. M. (1995). Chromosome condensation induced by fostriecin does not require p34*^cdc2^* kinase activity and histone H1 hyperphosphorylation, but is associated with enhanced histone H2A and H3 phosphorylation. *EMBO J.* 14 976–985. 10.1002/j.1460-2075.1995.tb07078.x 7889943 PMC398169

[B25] Honkanen R. E. Codispoti B. A. Tse K. Boynton A. L. (1994). Characterization of natural toxins with inhibitory activity against serine-threonine protein phosphatases. *Toxicon* 32 339–350. 10.1016/0041-0101(94)90086-8 8016855

[B26] Honkanen R. E. Zwiller J. Moore R. E. Daily S. L. Khatra B. S. Dukelow M. (1990). Characterization of microcystin-LR, a potent inhibitor of type 1 and type 2A protein phosphatases. *J. Biol. Chem.* 265 19401–19404. 2174036

[B27] Houben A. Demidov D. Caperta A. D. Karimi R. Agueci F. Vlasenko L. (2007). Phosphorylation of histone H3 in plants - a dynamic affair. *Biochim. Biophys. Acta* 1769 308–315. 10.1016/j.bbaexp.2007.01.002 17320987

[B28] Hsu J. Y. Sun Z. W. Li X. Reuben M. Tatchell K. Bishop D. K. (2000). Mitotic phosphorylation of histone H3 is governed by Ipl1/aurora kinase and Glc7/PP1 phosphatase in budding yeast and nematodes. *Cell* 102 279–291. 10.1016/S0092-8674(00)00034-9 10975519

[B29] Kós P. Gorzó G. Surányi G. Borbely G. (1995). Simple and efficient method for isolation and measurement of cyanobacterial hepatotoxins by plant tests (*Sinapis alba* L.). *Anal. Biochem.* 225 49–53. 10.1006/abio.1995.1106 7778786

[B30] Li M. Makkinje A. Damuni Z. (1996). The myeloid leukemia-associated protein SET is a potent inhibitor of protein phosphatase 2A. *J. Biol. Chem.* 271 11059–11062. 10.1074/jbc.271.19.11059 8626647

[B31] Luan S. (2003). Protein phosphatases in plants. *Annu. Rev. Plant Biol.* 54 63–92. 10.1146/annurev.arplant.54.031902.134743 14502985

[B32] MacKintosh C. Beattie K. A. Klumpp S. Cohen P. Codd G. A. (1990). Cyanobacterial microcystin-LR is a potent and specific inhibitor of protein phophatases 1 and 2A from both mammals and higher plants. *FEBS Lett.* 264 187–192. 10.1016/0014-5793(90)80245-E 2162782

[B33] MacKintosh C. Diplexcito J. (2003). *Naturally Occurring Inhibitors of Serine/Threonine Phosphatases, in Handbook of Cell Signaling* Vol. 1. USA: Elsevier Science Publisher 607–611. 10.1016/B978-0-12-374145-5.X0001-0

[B34] Manzanero S. Rutten T. Kotseruba V. Houben A. (2002). Alterations in the distribution of histone H3 phosphorylation in mitotic plant chromosomes in response to cold treatment and the protein phosphatase inhibitor cantharidin. *Chromosome Res.* 10 467–476. 10.1023/A:1020940313841 12489829

[B35] Máthé C. Beyer D. Erdődi F. Serfőző Z. Székvölgyi L. Vasas G. (2009). Microcystin-LR induces abnormal root development by altering microtubule organization in tissue-cultured common reed (*Phragmites australis*) plantlets. *Aquat. Toxicol.* 92 122–130. 10.1016/j.aquatox.2009.02.005 19269700

[B36] Máthé C. Beyer D. M-Hamvas M. Vasas G. (2016). The effects of microcystins (cyanobacterial heptapeptides) on the eukaryotic cytoskeletal system. *Mini Rev. Med. Chem.* 16 1063–1077. 10.2174/1389557516666160219130732 26891927

[B37] Máthé C. Vasas G. Borbély G. Erdődi F. Beyer D. Kiss A. (2013). Histological, cytological and biochemical alterations induced by microcystin-LR and cylindrospermopsin in white mustard (*Sinapis alba* L.) seedlings. *Acta Biol. Hung.* 64 75–89. 10.1556/ABiol.64.2013.1.7 23567832

[B38] Murashige T. Skoog F. (1962). A revised medium for rapid growth and bioassays with tobacco tissue cultures. *Physiol. Plantarum* 15 473–497. 10.1111/j.1399-3054.1962.tb08052.x

[B39] Nemani R. Lee E. Y. C. (1993). Reactivity of sulfhydryl groups of the catalytic subunits of rabbit skeletal muscle protein phosphatases 1 and 2A. *Arch. Biochem. Biophys.* 300 24–29. 10.1006/abbi.1993.1004 8380964

[B40] Nguyen N. M. Gonda S. Vasas G. (2013). A review on the phytochemical composition and potential medicinal uses of horseradish (*Armoracia rusticana*) root. *Food Rev. Int.* 29 261–275. 10.1080/87559129.2013.790047

[B41] Olszewska M. J. Marciniak K. Kuran H. (1990). The timing of synthesis of proteins required for mitotic spindle and phragmoplast in partially synchronized root meristems of vicia faba L. *Eur. J. Cell Biol.* 53 89–92. 2076711

[B42] Øverby A. Stokland R. A. Åsberg S. E. Sporsheim B. Bones A. M. (2015). Allyl isothiocyanate depletes glutathione and upregulates expression of glutathione S-transferases in *Arabidopsis thaliana*. *Front. Plant Sci.* 6:277. 10.3389/fpls.2015.00277 25954298 PMC4406002

[B43] Pan W. H. Houben A. Schegel R. (1993). Highly effective cell synchronization of plant roots by hydroxylurea and amiprophos-methyl or colchicine. *Genome* 36 387–390. 10.1139/g93-053 18469996

[B44] Pereira S. R. Vasconcelos V. M. Antunes A. (2011). Computational study of the covalen bonding of microcystins to cysteine residues- a reaction involved in the inhibition of the PPP family of protein phosphatases. *FEBS J.* 280 674–680. 10.1111/j.1742-4658.2011.08454.x 22177231

[B45] Pflugmacher S. (2016). Biotransformation of microcystins in eukaryotic cells- possible future research directions. *Mini Rev. Med. Chem.* 16 1078–1083. 10.2174/1389557516666160219130837 26891926

[B46] Pinsky B. A. Nelson C. R. Biggins S. (2009). Protein phosphatase 1 regulates exit from the spindle checkpoint in budding yeast. *Curr. Biol.* 19 1182–1187. 10.1016/j.cub.2009.06.043 19592248 PMC2731492

[B47] Purhonen A. K. Louhivouri L. M. Kiehne K. Åkerman K. E. O. Herzig K. H. (2008). TRPA1 channel activation induces cholecystokinin release via extracellular calcium. *FEBS Lett.* 582 229–232. 10.1016/j.febslet.2007.12.005 18082143

[B48] Schroeder-Reiter E. Houben A. Wanner G. (2003). Immunogold labeling of chromosomes for scanning electron microscopy: a closer look at phosphorylated histone H3 in mitotic metaphase chromosomes of *Hordeum vulgare*. *Chromosome Res.* 11 585–596. 10.1023/A:1024952801846 14516067

[B49] Shi Y. (2009). Serine/threonine phosphatases: mechanism through structure. *Cell* 30 468–484. 10.1016/j.cell.2009.10.006 19879837

[B50] Shima H. Tohda H. Aonuma S. Nakayasu M. DePaoli-Roach A. A. Sugimura T. (1994). Characterization of the PP2Aa gene mutation in okadaic acid-resistant variants of CHO-K1 cells. *Proc. Natl. Acad. Sci. U.S.A.* 91 9267–9271. 10.1073/pnas.91.20.9267 7937753 PMC44793

[B51] Smith T. K. Lund E. K. Parker M. L. Clarke R. G. Johnson I. T. (2004). Allyl-isothiocyanate causes mitotic block, loss of cell adhesion and disrupted cytoskeletal structure in HT29 cells. *Carcinogenesis* 25 1409–1415. 10.1093/carcin/bgh149 15033907

[B52] Spinner L. Gadeyne A. Belcram K. Goussot M. Moison M. Duroc Y. (2013). A protein phosphatase 2A complex spatially controls plant cell division. *Nat. Commun.* 4:1863. 10.1038/ncomms2831 23673648

[B53] Takemiya A. Ariyoshi A. Shimazaki K. (2009). Identification and functional characterization of Inhibitor-3, a regulatory subunit of protein phosphatase 1 in plants. *Plant Physiol.* 150 144–156. 10.1104/pp.109.135335 19329567 PMC2675749

[B54] Templeton G. W. Nimick M. Morrice N. Campbell E. Goudreault M. Gingras A.-C. (2011). Identification and cheracterization of Atl-2, an *Arabidopsis* homologue of an ancient protein phosphatase 1 (PP1) regulatory subunit. *Biochem. J.* 435 73–83. 10.1042/BJ20101035 21222654

[B55] Teruya T. Simizu S. Kanoh N. Osada H. (2005). Phoslactomycin targets cysteine-269 of the protein phosphatase 2A catalytic subunits in cells. *FEBS Lett.* 579 2463–2468. 10.1016/j.febslet.2005.03.049 15848189

[B56] Tunquist B. J. Maller J. L. (2003). Under arrest: cytostatic factor (CSF)-mediated metaphase arrest in vertebrate eggs. *Genes Dev.* 17 638–710. 10.1101/gad.1071303 12651887

[B57] Uhrig R. G. Labandera A.-M. Moorhead G. B. (2013). Arabidopsis PPP family of serine/threonine protein phosphatases: many targets but few engines. *Trends Plant Sci.* 18 505–513. 10.1016/j.tplants.2013.05.004 23790269

[B58] Vasas G. Gáspár A. Páger C. Surányi G. Máthé C. M-Hamvas M. (2004). Analysis of cyanobacterial toxins (anatoxin-a, cylindrospermopsin, microcystin-LR) by capillary electrophoresis. *Electrophoresis* 25 108–115. 10.1002/elps.200305641 14730574

[B59] Virshup D. M. Shenolikar S. (2009). From promiscuity to precision: protein phosphatases get a makeover. *Mol. Cell* 33 537–545. 10.1016/j.molcel.2009.02.015 19285938

[B60] Yadav V. Gao X.-H. Willard B. Hatzoglou M. Banerjee R. Kabil O. (2017). Hydrogen sulfide modulates eukaryotic translation initiation factor 2α (eIF2α) phosphorylation status in the integrated stress-response pathway. *J. Biol. Chem.* 292 13143–13153. 10.1074/jbc.M117.778654 28637872 PMC5555178

[B61] Zhang K. Tsukitani Y. John P. C. L. (1992). Mitotic arrest in tobacco caused by the phosphoprotein phosphatase inhibitor okadaic acid. *Plant Cell Physiol.* 33 677–688. 10.1093/oxfordjournals.pcp.a078305

[B62] Zhang Y. (2010). Allyl isothiocyanate as a cancer chemopreventive phytochemical. *Mol. Nutr. Food Res.* 54 127–135. 10.1002/mnfr.200900323 19960458 PMC2814364

