# Supplementary figures and images for: Allyl-Isothiocyanate and Microcystin-LR Reveal the Protein Phosphatase Mediated Regulation of Metaphase-Anaphase Transition in Vicia faba
Source: Front Plant Sci. 2018 Dec 13;9:1823. doi: 10.3389/fpls.2018.01823 (PMC6300510; doi:10.3389/fpls.2018.01823)

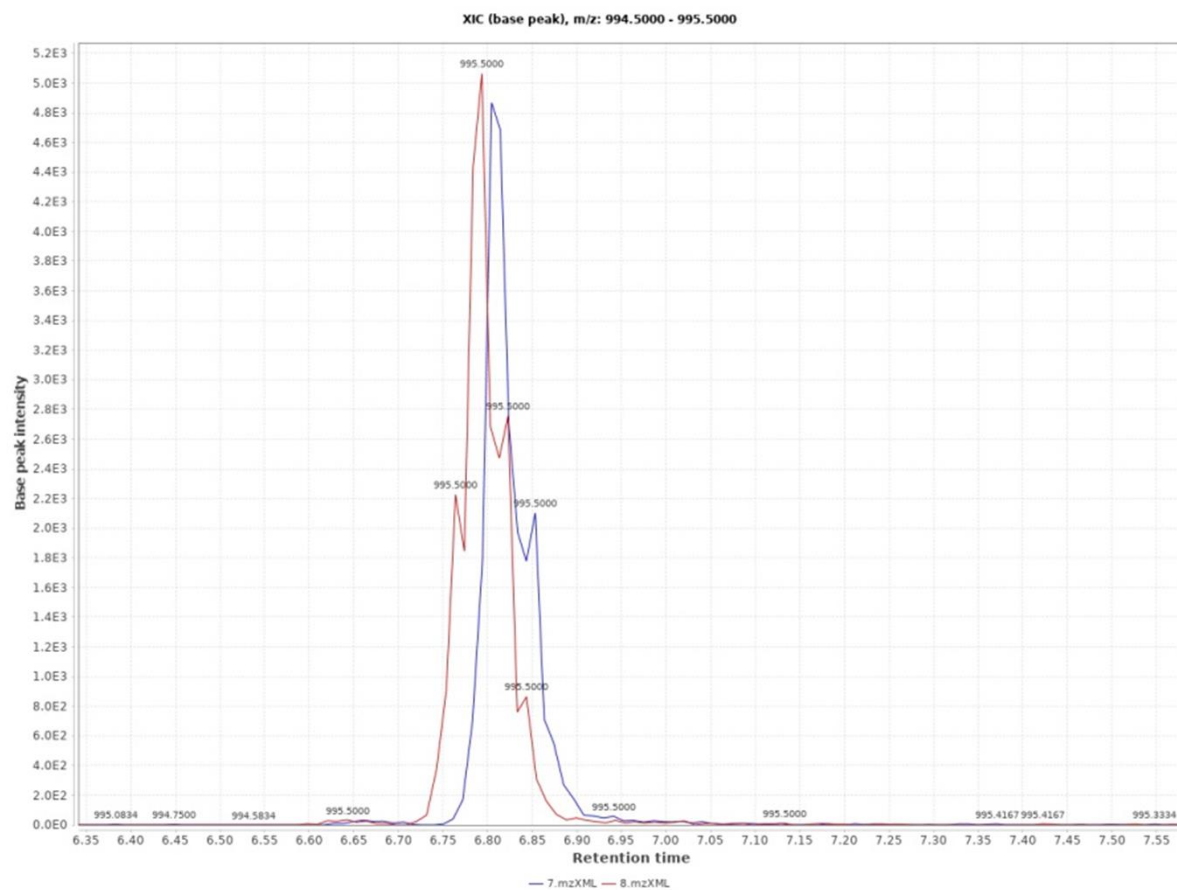

Fig. S1

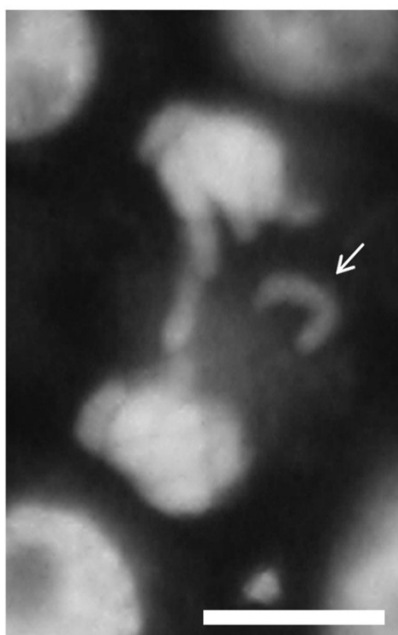

Fig. S2

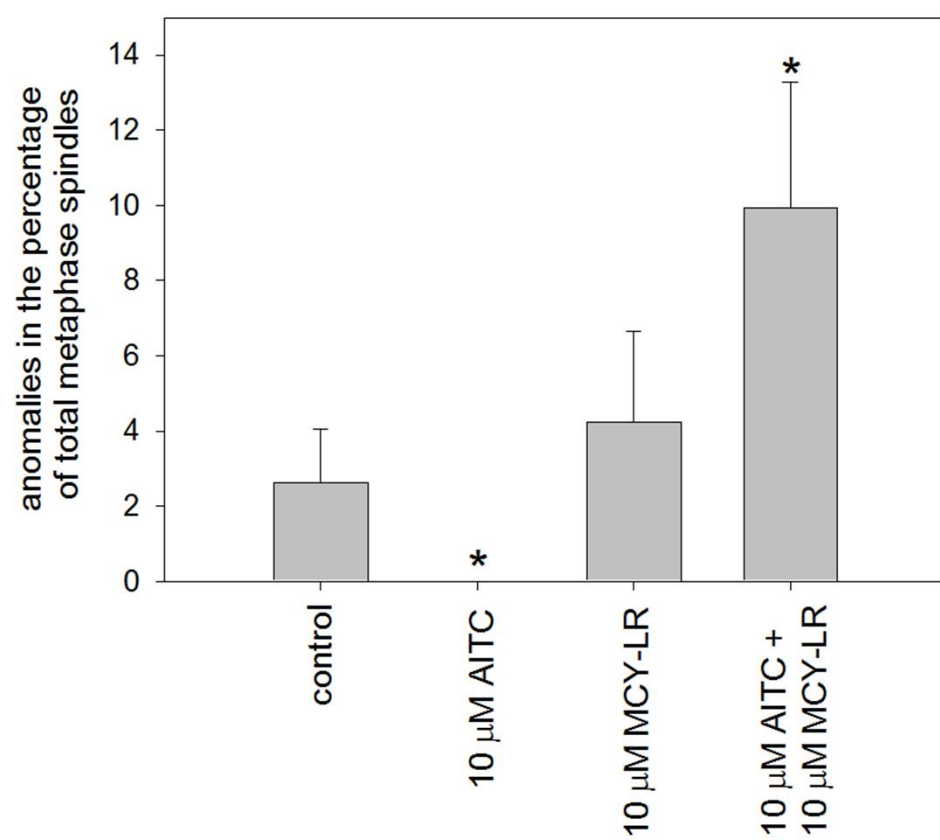

Fig. S3

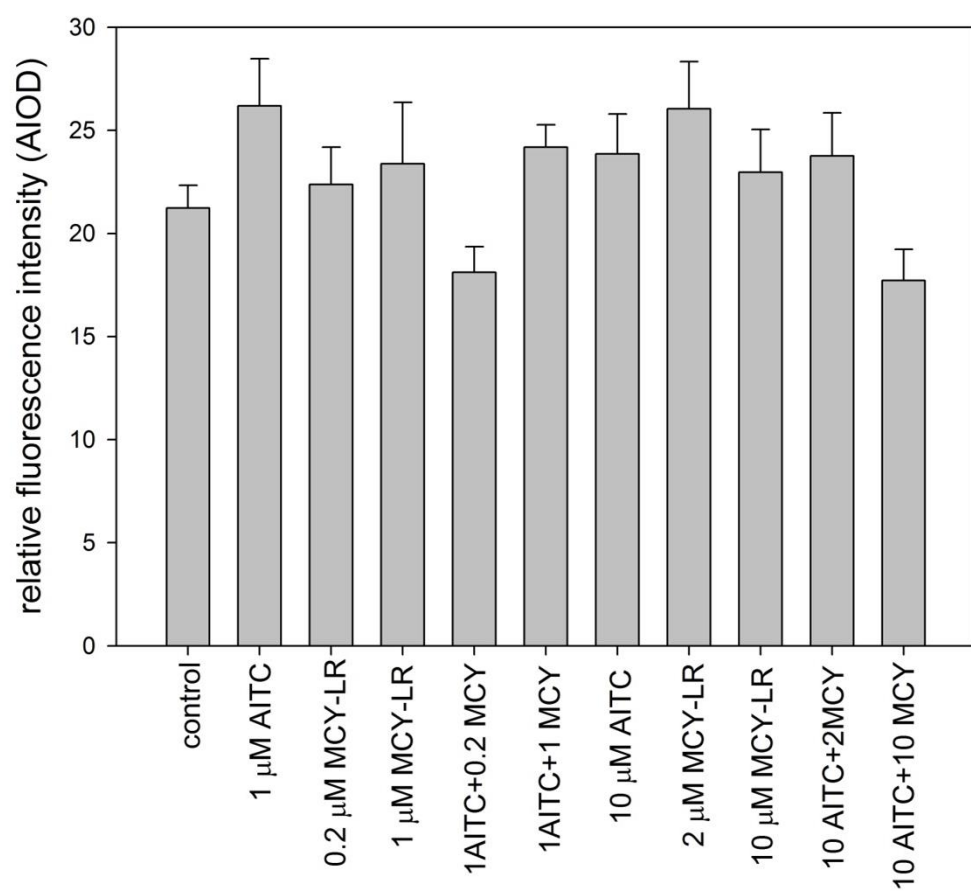

Fig. S4

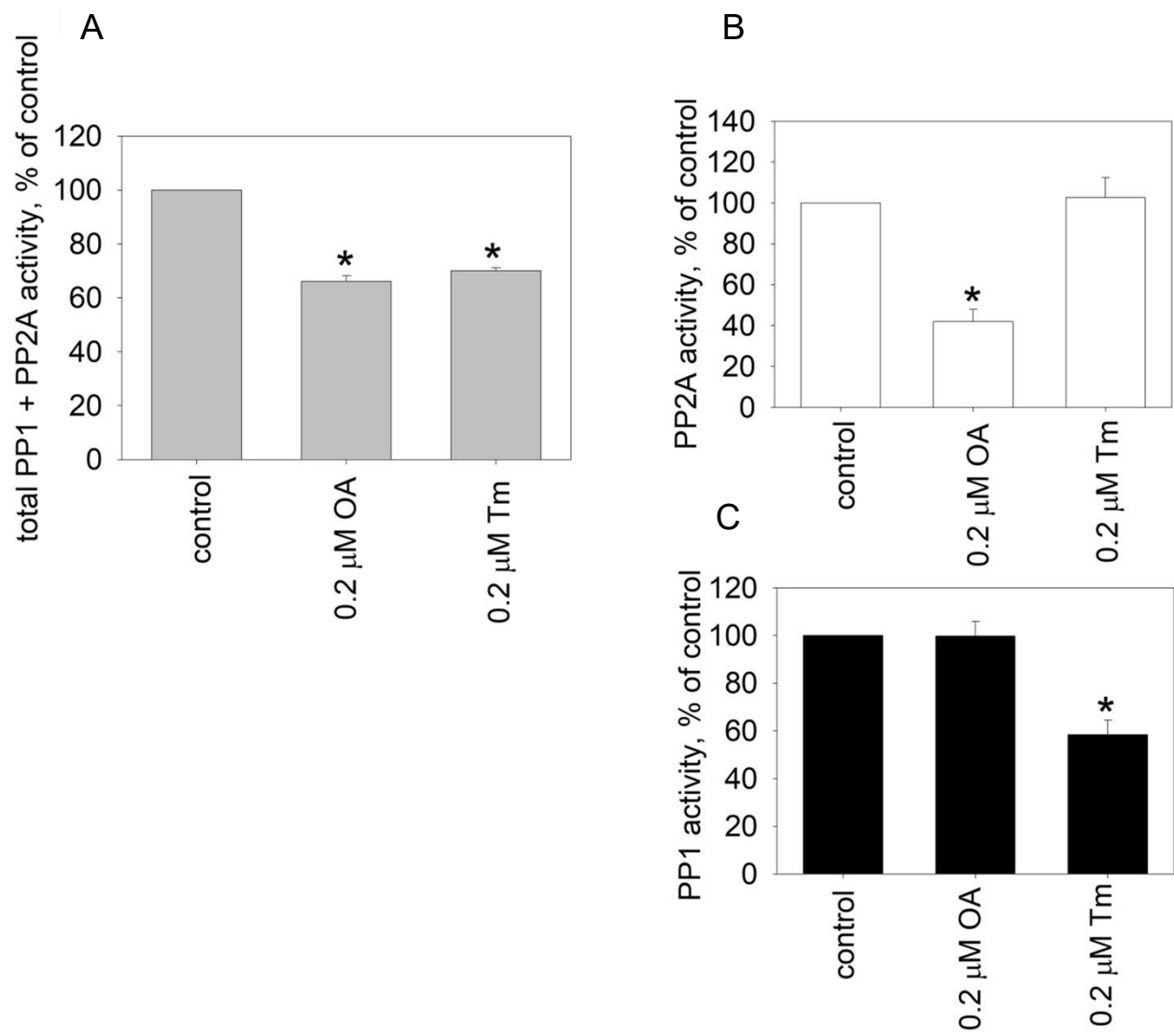

Fig. S5

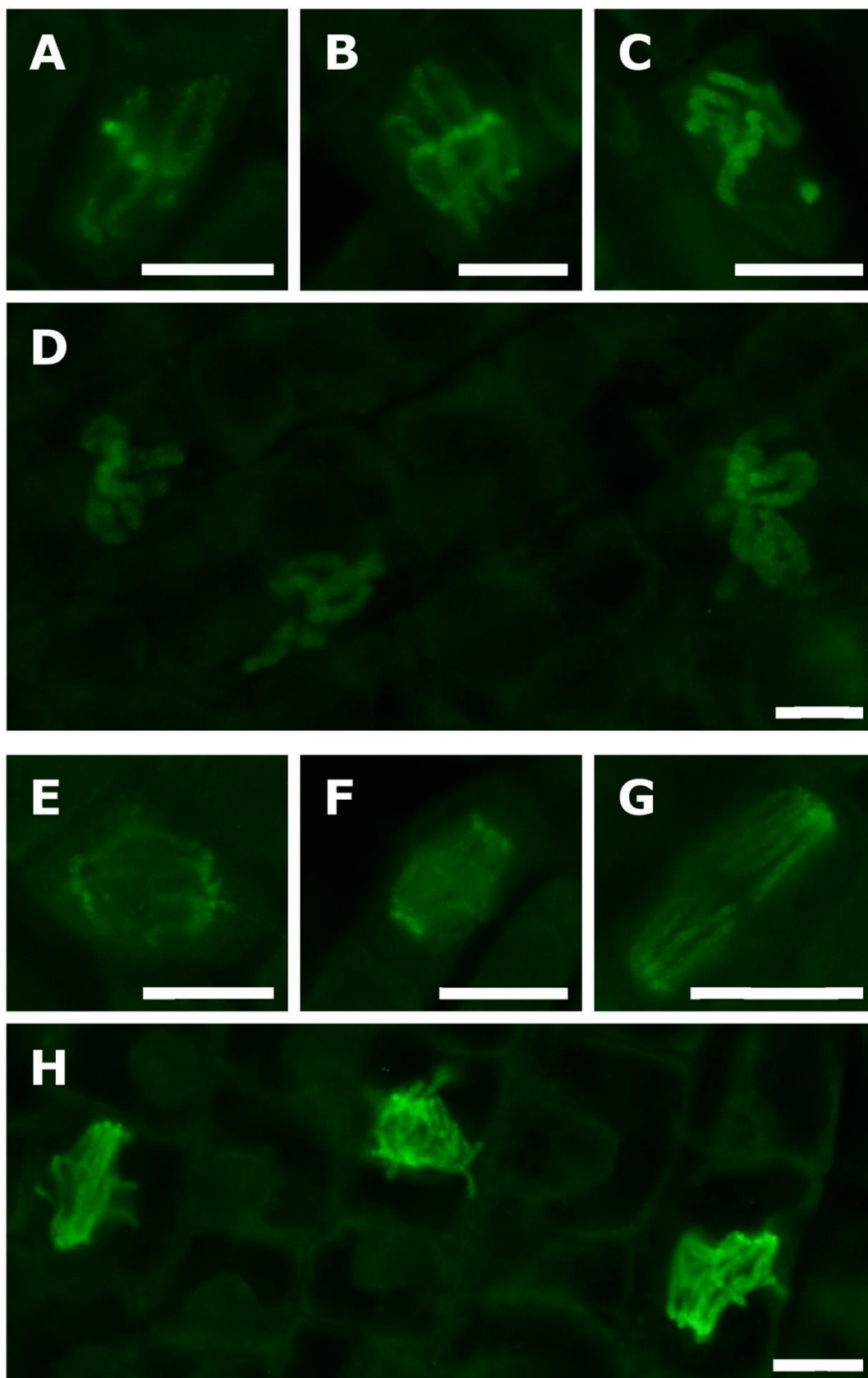

Fig. S6

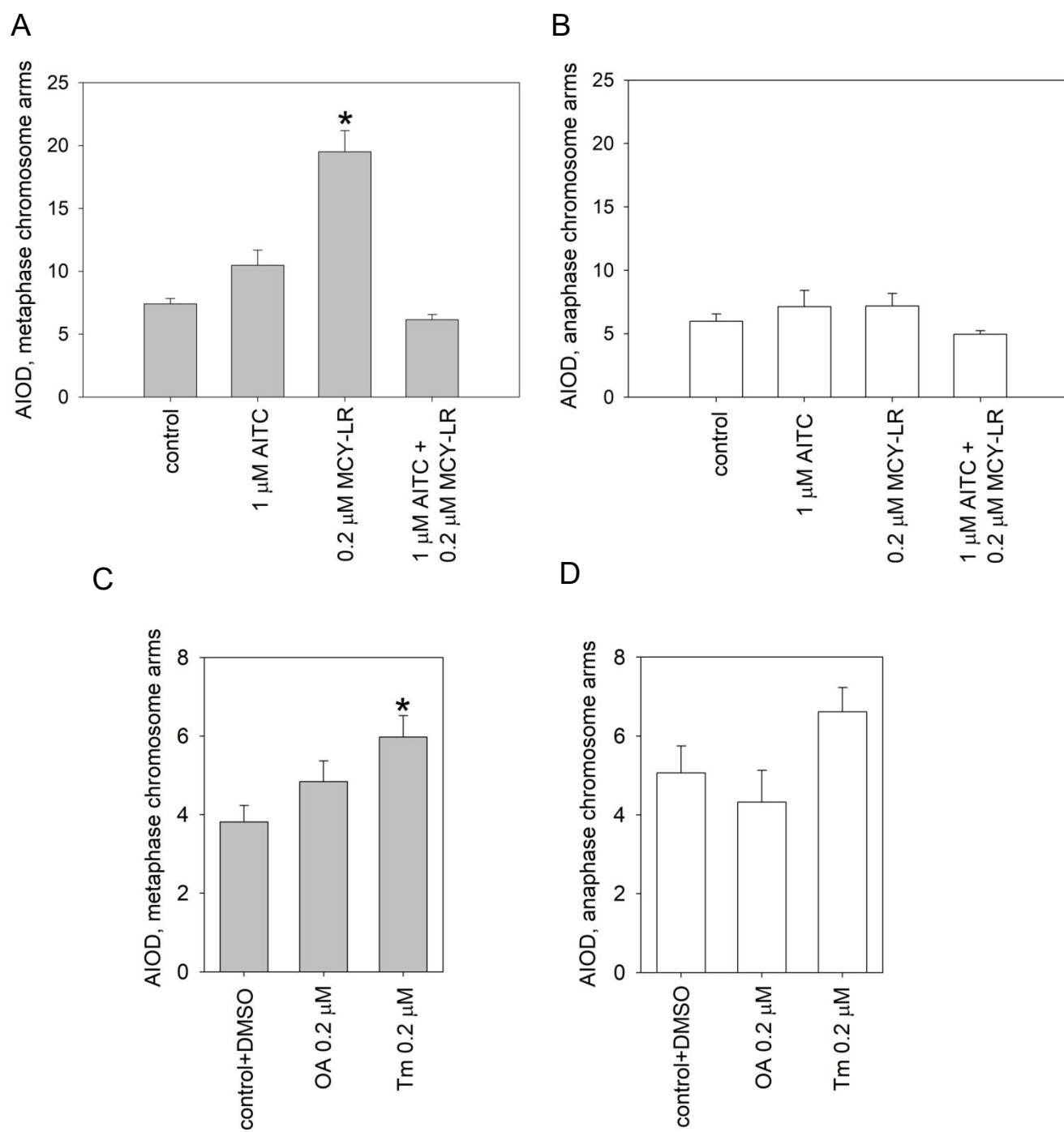

Fig. S7

Supplement: FIGURE S1 — LC-MS spectrogram of a desalted, sucrose-free liquid MS medium containing 10 μM MCY-LR (red peak) and 10 μM AITC + μM MCY-LR (blue peak) shows no significant difference between the mass spectra. [file Data_Sheet_1.pdf]
